# Endothelial shear stress enhancements: a potential solution for critically ill Covid-19 patients

**DOI:** 10.1186/s12938-020-00835-7

**Published:** 2020-12-03

**Authors:** Sayed Nour

**Affiliations:** Le LAB’O, Orleans Technopole, 1 avenue du Champs de Mars, 45074 Orleans, France

**Keywords:** Acute respiratory distress syndrome, Mechanical ventilation, Endothelial shear stress, Multiple organ failure, Cardiac arrest

## Abstract

Most critically ill Covid-19 patients succumb to multiple organ failure and/or sudden cardiac arrest (SCA) as a result of comorbid endothelial dysfunction disorders which had probably aggravated by conventional mechanical assist devices. Even worse, mechanical ventilators prevent the *respiratory pump* from performing its crucial function as a potential generator of endothelial shear stress (ESS) which controls microcirculation and hemodynamics since birth. The purpose of this work is to bring our experience with ESS enhancement and pulmonary vascular resistance (PVR) management as a potential therapeutic solution in acute respiratory distress syndrome (ARDS). We propose a non-invasive device composed of thoracic and infradiaphragmatic compartments that will be pulsated in an alternating frequency (20/40 bpm) with low-pressure pneumatic generator (0.1–0.5 bar). Oxygen supply, nasogastric with, or without endotracheal tubes are considered.

## Background

“The great tragedy of Science is the slaying of a beautiful hypothesis by an ugly fact” Thomas Huxley [1].

Severe acute respiratory syndrome (SARS) has become a global healthcare issue over the last two decades [2]. Mechanical ventilation and pharmacological supports (e.g., vasopressors) are the conventional treatment for those who develop acute respiratory distress syndrome (ARDS). Even though a number of devices and means adjunctive to conventional ARDS therapy, e.g., extracorporeal membrane oxygenation (ECMO), artificial kidney, the survival rate still remains quite poor [3]. As many predictions and controversies are still surrounding Covid-19 management, the disease has claimed more than one and a quarter million lives around the Globe and remains significantly contagious, regardless of healthcare progress. Thus, fundamental and out-of-scope research is required in nearly all aspects of ARDS management.

## Endothelial dysfunction disorders in Covid-19 patients

Knowing that the Covid-19 virus invades host cells via the angiotensin-converting enzyme receptor 2 (ACE2) [4], yet most complications and deaths occur as a result of endothelial dysfunction disorders, whether in the form of comorbid conditions, e.g., arterial hypertension, mediated by pathogens, e.g., inflammatory response, and/or iatrogenic due to current therapies, e.g., thromboembolic syndrome [5]. As a reminder, endothelial shear stress (ESS) controls and maintains endothelial functions, e.g., vascular tone, coagulation, angiogenesis, apoptosis, diabetes, atherosclerosis, immune system, inflammatory response, nitric monoxide synthesis, etc., [6]. Also, ESS controls vasculogenesis, cardiogenesis, embryogenesis, and organogenesis through the angiogenesis-apoptosis interdependency process, from the 8th day of gestation until death. An imbalanced angiogenesis-apoptosis interdependency can induce irreversible cellular damages, like Eisenmenger syndrome, Cor pulmonale, or heart failure: cardiomyocytes apoptosis, compensated by angiogenic hypertrophy or fibrotic dilatation [7]. In other words, remodelling is a time-consuming process, which refutes claims of acute myocardial dilation in Covid-19 patients [8].

## ESS-inducing circulatory driving forces

**Fig. 1:**
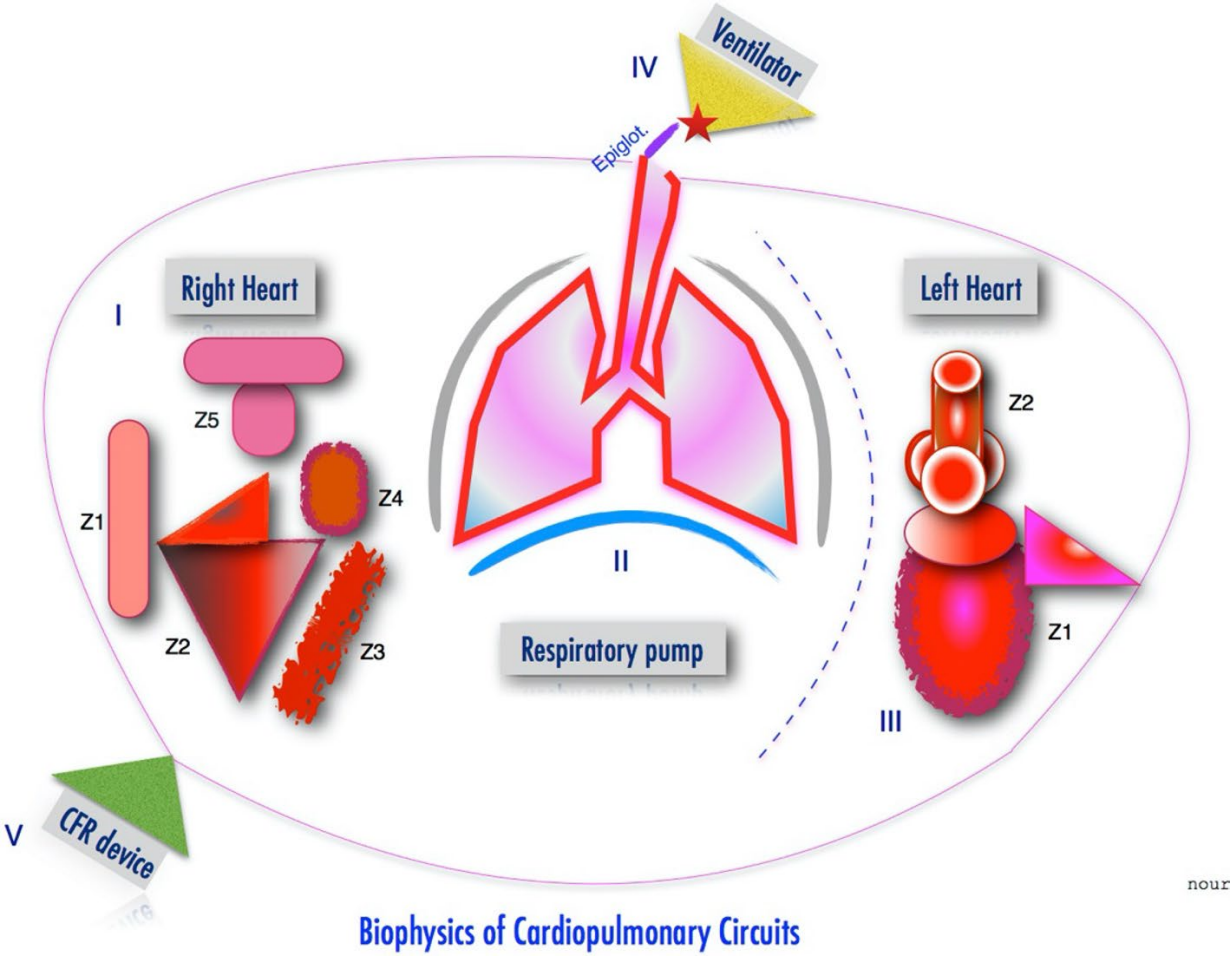
Biophysics of cardiopulmonary circulatory driving forces. I Right-heart circuit main remodeling zones: Z1, systemic veins; Z2, right ventricle; Z3, septum; Z4, infundibulum; Z5, pulmonary artery [10]. II Respiratory pump, a low-pressure, momentarily hydraulic circuit that compresses two types of fluids: Newtonian (air) and non-Newtonian (blood), in an accordion-like manner, creating ESS. III Left-heart circuit main remodeling zones: Z1, left ventricle; Z2, Aorta and Valsalva. IV Mechanical ventilator. V: CFR device [23]

In the antenatal period, the right ventricle (RV) is the main trigger of ESS and moderator of fetal development, even in severe cardiomyopathies, e.g., hypoplastic left heart syndrome. It distributes blood flow to the left ventricle (LV) through the foramen ovale and the descending aorta through the ductus arteriosus, while the pulmonary artery (PA) with collapsed lungs receives about 10% of blood volume (BV). In the postnatal period (Fig. 1), after shunts closures, while LV and peristaltic arteries represent the main circulatory driving forces, at the left-heart side that contains less than 10% of BV [9], the respiratory pump becomes a key circulatory driving force to deal with the massive BV at the right-heart side (≥ 70%). The respiratory pump is a low-pressure momentarily closed hydraulic circuit, due to the epiglottis effect, must deal with two types of fluids: the compressible Newtonian (air) and the incompressible non-Newtonian (blood), and a delicate alveolar system is composed of two types of single-cell layers: the epithelium and the endothelium, to ensure gas exchanges. It becomes the main trigger of ESS to continue cardiovascular remodeling, e.g., increasing LV mass in maintaining low remodeling at the right-heart side, e.g., RV/LV mass ≈ 1/6. It squeezes the pulmonary parenchyma in an *accordion-like* manner, to release plenty of endothelial mediators to drop the pulmonary vascular resistances (PVR), to improve hemodynamics as well as tissue oxygenation with first breath after birth. By controlling the pulmonary afterload, the respiratory pump controls RV preload and cardiac output (Frank–Starling law), helped with other influential forces, like the muscle pumps, gravity, and atmospheric pressure [10]. Functionally, the respiratory pump can redress hemodynamics and remedy the side effects of endothelial dysfunction caused by conventional circulatory assist devices (CAD), which may explain long-term survival of continuous flow artificial-hearts transplants [11]. Also, an underdeveloped *respiratory pump* can explain failure of right heart bypass procedures in very young age [12].

## Disadvantages of current ARDS management

The therapeutic goal in a critically ill patient is to improve hemodynamics and tissue oxygenation in order to maintain healthy cellular metabolism to promote rapid recovery with restoring endothelial functions, e.g., angiogenesis-apoptosis interdependency [13]. Meanwhile, once vital metabolic processes are threatened, regardless of the underlying pathology, it becomes a matter of resistance and fluid mechanics management. As it is known, the human being is a multicellular organism in which cell biology plays a main role in terms of development, maintenance, proper functioning, and also failure of vital organs [14]. Maintaining good metabolic processes depends on organs’ microcirculation which is controlled by plurality of endothelial mediators of vasodilators induced by ESS [15, 16]. However, relying on *systemic afterload* to improve hemodynamics may worsen endothelial dysfunction conditions, such as vasopressors that increase vascular resistance and myocardial oxygen consumption, most likely end in organ failure and circulatory assist devices (CAD) requirement. Yet, the ugly fact is that the important role of the *respiratory pump* as a master-key circulatory driving force and a potential generator of ESS is still disregarded by therapists. As ventilators with endotracheal intubation and neuromuscular blockades transform the *respiratory pump* into a piston-like, closed, pressurized, immobilized, (purulent), hydraulic circuit, along with their interference with coronary perfusion flow are the principle causes of hemodynamic and metabolic deterioration in ARDS [17, 18]. In addition, the extra-alveolar and alveolar endothelial cells of the fragile alveolar system are embryologically different, which promotes serious complications, such as insufficient inhalational pulmonary arterial hypertension (PAH) therapies, barotrauma, and most likely alveolar fibrosis [19, 20]. Besides, the lack of influential circulatory driving forces, like respiratory and muscle pumps and gravity, in bedridden ventilated patients severely disturbs RV preload, promoting further hemodynamic deterioration and serious endothelial dysfunction conditions, e.g., thromboembolic syndrome. Moreover, the encounter of CAD with the circulatory system creates vicious circle of endothelial dysfunction and momentum energy losses, e.g., boundary wall friction of bloodstream inside rigid narrow conduits of CAD promotes hemorrhage, thromboembolism, inflammatory response, apoptosis, etc., until multiple organ failure. Likewise, pressurized airflow inside ventilators rigid conduits but with different diameters promotes barotrauma and surinfection by Venturi effect.

Alternatively, we propose ESS enhancement therapy as a potential solution in ARDS. Based on our previous experience with ESS enhancement therapy, we have proved that *pulmonary afterload* controls *systemic afterload* and hemodynamics in pediatric models of cardiogenic shock [21]. The proposed concept was presented at the American Thoracic Society conference in 2014 [22], and based on a *circulatory flow restoration* device, tested in pediatric models with sudden cardiac arrest [23]. Our main goal is to develop a low-pressure non-invasive cardiopulmonary circulatory assist device capable of maintaining a full function of the *respiratory pump* to improve organs’ perfusion–oxygenation and promote patients’ recovery in better metabolic and hemodynamic conditions.

## Methods

### Principles of ESS therapy

The clinical applications of ESS with CAD are controlled by several diversities between the cardiovascular system and lumped models [24]. As lumped models are constructed for driving a Newtonian compressible fluid inside a closed pressurized hydraulic circuit, implementing rigid tubes with fixed diameter [25], in practices a CAD is confronted with a non-Newtonian fluid (blood), running in flexible vessels with different geometries, which creates a vicious circle of momentum energy losses and endothelial dysfunction, manifested clinically with the postcardiotomy syndrome [26] and post-hemodialysis pains [27]. Therefore, a CAD for ARDS management must adapt to the pathophysiology and biophysics of the cardiopulmonary—circulatory system (Fig. 1), maintain a fully functional *respiratory pump,* and avoid the confrontation of opposing hydraulic circuits.

### Device

**Fig. 2:**
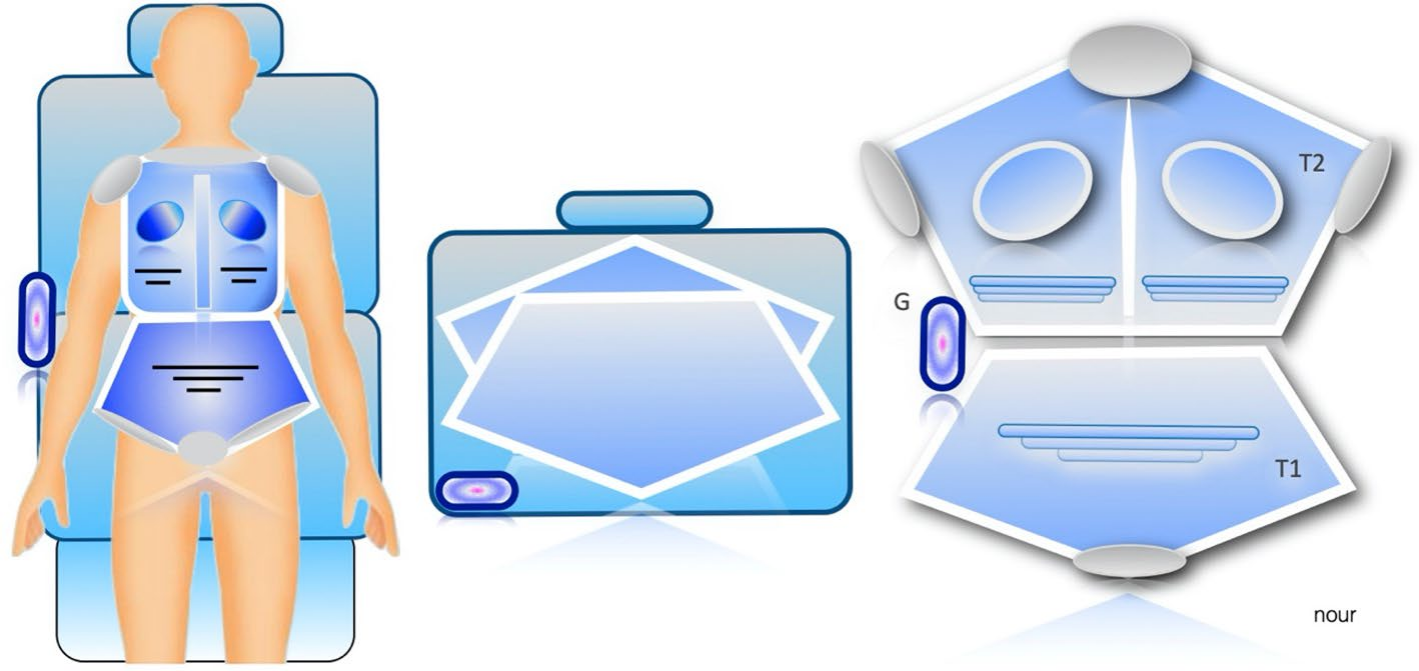
Middle panel: showing a CFR device suitcase. Right panel: showing a Vest (T2) and Corset (T1) and Console of commands (G). Left panel: showing a schema of wrapped CFR device around a patient on a deployed medical stretcher suitcase

**Fig. 3:**
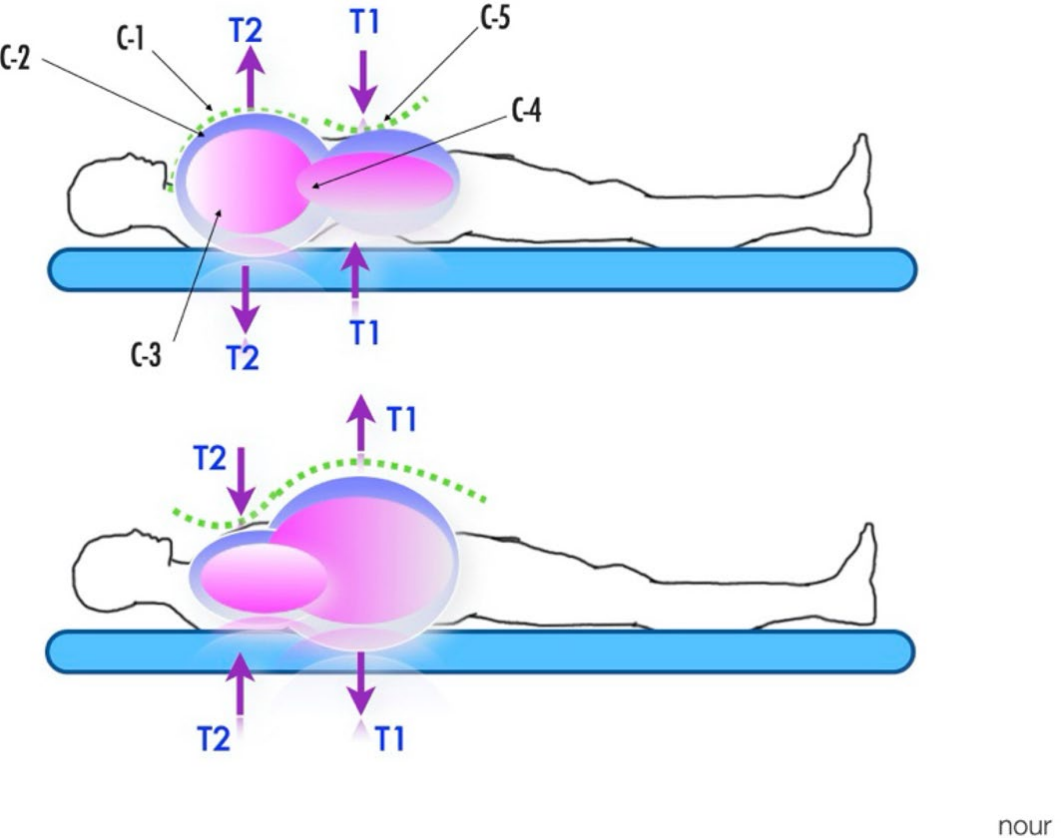
A schema representing the mechanism of the alternating pulsations of the thoracic (T2) and abdominal (T1) compartments of the CFR device. The upper panel shows the CFR in an inspiration phase. The lower panel shows the CFR in an expiration phase. C = covering endothelial layer shearing zone; C-1 = thoracic cage; C-2 = mediastinal shearing mass; C-3 = pulmonary parenchyma, C-4 = diaphragmatic pump; C-5 = hepatosplanchnic shearing mass

As represented in Fig. 2, a non-invasive low-pressure pulsatile device, composed of an infradiaphragmatic (Corset) and supradiaphragmatic (Vest) compartments, commanded by an electro-mechanic pneumatic generator^1^. In general, the chosen materials and design must adapt to different genders and body sizes to allow wrapping of the device around the patient’s body rapidly and tightly by therapists. Once wrapped around the patient’s body, the switched-on generator will induce alternating pulsations at the trunk and chest compartments (Fig. 3), in a low pressure (e.g., 0.1–0.5 bars) and a fixed frequency of 40 bpm, during cardiac arrest. In case of return of heartbeat, the device frequency will be reduced to one-in-two mode (Vest/Corset: 20/40 bpm) to avoid undesired results, e.g., parenchymal injuries. Likewise, the one-in-two mode will be selected for patients’ ventilation, e.g., ARDS. The oxygen supply means will be determined according to patients’ conditions, e.g., as a non-invasive ventilator (NIV) or invasively with endotracheal intubation to allow bronchioalveolar lavage in severe cases. In either condition, the device will not restrict the mobility of the chest wall. Besides, the automated device set allows at-ease patients’ inclination as well as for additional medical instrumentations, if requested. A nasogastric tube must be considered.

## Proof of concept

### Objectives

The objective of this study was to improve restoration and maintenance of circulatory flow dynamics with ESS and creating a nearly physiological arterial pressure curve (systolic ≥ 80 mmHg) to promote adequate vital organs’ perfusion, regardless of heartbeat.

### Study design

Two pediatric models with different cardiotorsal anatomies were selected for this study. For humanitarian reasons, we have restricted the animals’ number in the cardiopulmonary resuscitation (CPR) group to avoid the well-known unnecessary animal losses [28], and also due to the impossibility to obtain an appropriate arterial pressure curve with CPR for comparison. Therefore, we considered the first surviving animals of CPR for histopathological and serological comparative studies.

### Anesthesia

**Fig. 4:**
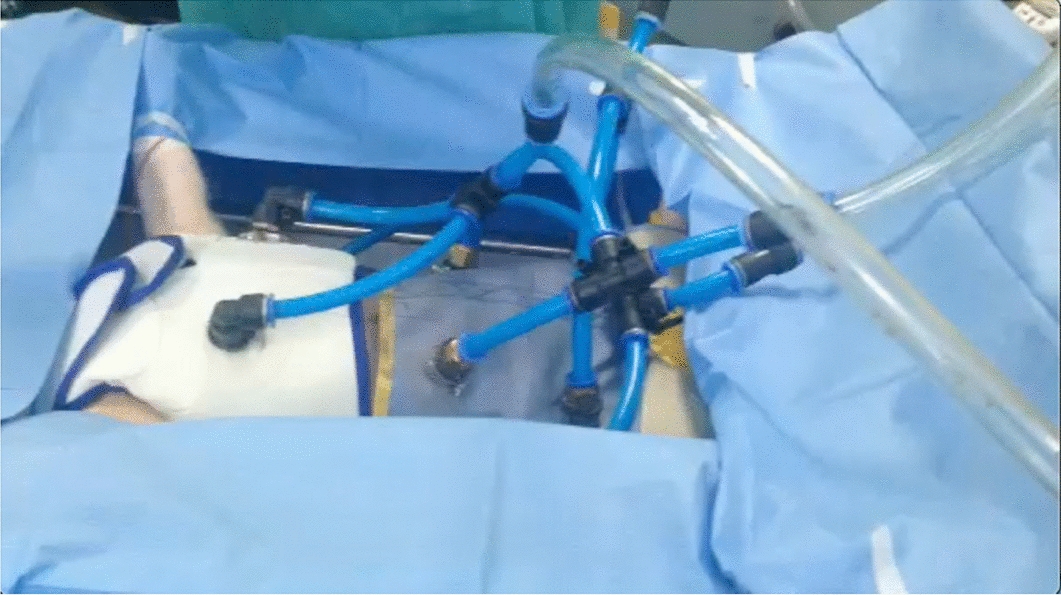
Thoracic vest and trousers prototype wrapped around piglet body and connected to pneumatic generator [23]

*Seven* domestic piglets of both sexes (6–8 kg) and *four* male pediatric dogs (7–10 kg) were premedicated with an anesthetic mixture composed of dihydroetorphine hydrochloride, dimethylaniline thiazole, ethylenediaminetetraacetic acid, and haloperidol, (3 mL) and midazolam (0.5 mg/kg), given intramuscularly; then prototypes were wrapped around the animal’s body, installed on a warmed operating table as shown in Fig. 4 and surveyed with a rectal probe (38 ± 1 °C). Anesthesia was maintained by 3% sodium phenobarbital (1 mg/kg), divided into doses and mechanical ventilation. Through a median cervicotomy and tracheotomy, a 3.5–5 # tracheal tube was inserted, followed by mechanical ventilation (PA-500 PuLang Technologies Inc®) with 40% oxygen, 10–15 mL/kg min tidal volume, and 15/min respiration frequency.

### Hemodynamic monitoring

The right carotid artery was isolated, and a 6F catheter was introduced. Then a Millar probe (4F MIKRO-TIP catheter transducer; Millar Instruments) was introduced through the carotid line into the aorta for continuous systemic arterial pressure (AP) monitoring (Biopac® physiology monitoring system). A 5F double-lumen central venous line (Hydrocath; BD Technologies) was introduced through the right internal jugular vein for central venous pressure monitoring and IV fluids. Cerebral blood flow was measured with a Transonic transit time flow meter (Transonic Systems, Ithaca, NY, USA), positioned around the left carotid artery. Urine output was measured by urinary catheter in dogs and direct suprapubic catheter insertion in piglets. Peripheral cutaneous microcirculation was measured by a laser flowmeter (Perimed PeriScan PIM 3 System) positioned at the earlobe (piglet) or tongue (dog).

### Procedures

Animals were subjected to different methods of sudden cardiac arrest (SCA): 10 mL (IV) of *potassium chloride* (KCl) (*n* = 7); *electric fibrillation* (*n* = 3); a 50 Hz and 50 V alternating current was delivered for 3 s through two acupuncture needles were inserted, one subcutaneously and one into the epicardium, connected to an external transthoracic alternating current defibrillator; and *asphyxia* by clamping the tracheal tube (*n* = 1). The cardiac arrest period was varied between 8 min (*n* = 2), 20 min (*n* = 3), and 30 min (*n* = 4). Two animals survived standard CPR after 8 min of cardiac arrest (e.g., cardiac massage-DC shock-adrenaline). The Corset/Vest prototypes were pulsated at a fixed alternating frequency of 40 bpm and in a low pressure of 0.01 MPa and 0.025 MPa, respectively. The ventilation was switched off after cardiac arrest and restarted in the 2 controls after return of heartbeat. The ventilator remained switched off with the tracheal tube connected to an oxygen bag in the entire treated group till the end of experiment.

### TUNEL test

The myocardium of the treated asphyxiated dog was harvested and compared to a dog that survived CPR after 8 min of cardiac arrest and kept alive for 6 h before to be euthanized with a 10 mL (IV) of KCl. The myocardial tissue was placed in 10% buffered formalin for 24 h, then mounted in paraffin and sectioned in 4 μm slices. The apoptotic cells were identified with a terminal deoxynucleotidyl transferase-mediated dUTP nick-end labeling (TUNEL) apoptosis detection kit according to the manufacturer’s protocol (Boster Inc, China). Five photographs (magnification 20×) were taken of each tissue section. All TUNEL-negative (blue) and TUNEL-positive (brown) nuclei were visualized under a light microscope; the total number of nuclei was counted in 5 random high-power fields from each sample. The apoptotic index (AI) was calculated as 100% * (TUNEL-positive nuclei/all nuclei).

### Serology test

Blood samples for S100 protein serology test as a detector of brain damage [29] were collected from the same two dogs, at baseline (T1) after cardiac arrest and before treatment (T2) and before sacrifice or by the end of experiment in the treated dog (T3). The S100 assay used ELISA kit (human S-100 (Soluble Protein-100) ELISA Kit, Elabscience biotechnology co., Ltd). An equation of standard curve for S100 is as follows: OD = 0.271 *C* + 0.011, where *C* (ng/mL) is the concentration of S100.

## Result

**Fig. 5:**
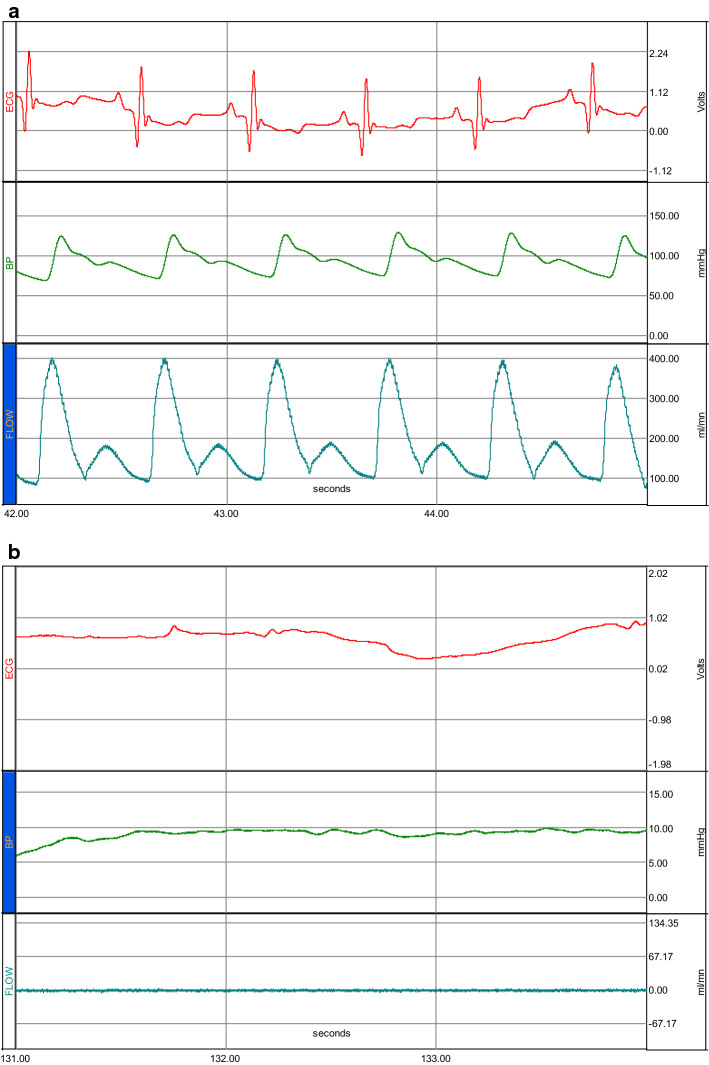

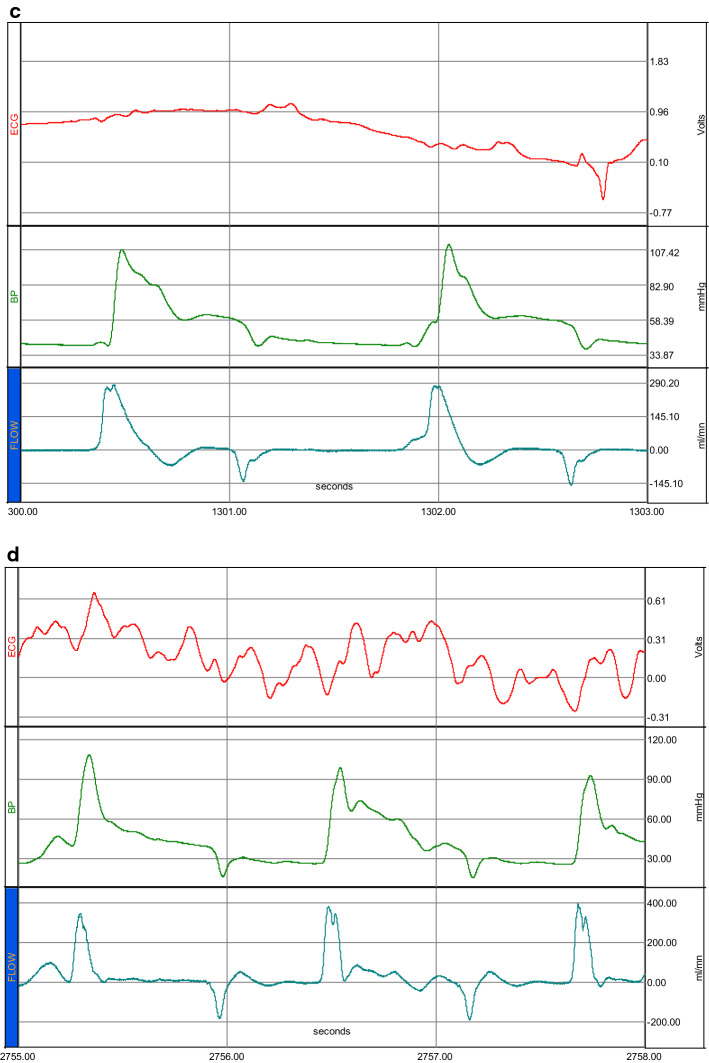
**a** Hemodynamic data obtained in a pediatric cardiac arrest model at baseline, showing ECG readings (upper line), aortic pressure line (middle curve), and carotid flow. **b** Post-cardiac arrest hemostatic data. **c** Restoration of hemodynamics with CFR device in a pediatric dog after 30 min of cardiac arrest by asphyxia [23]. **d** Restoration of hemodynamics with CFR device in a piglet after 20 min of cardiac arrest by 10 mL KCl (IV)

**Fig. 6:**
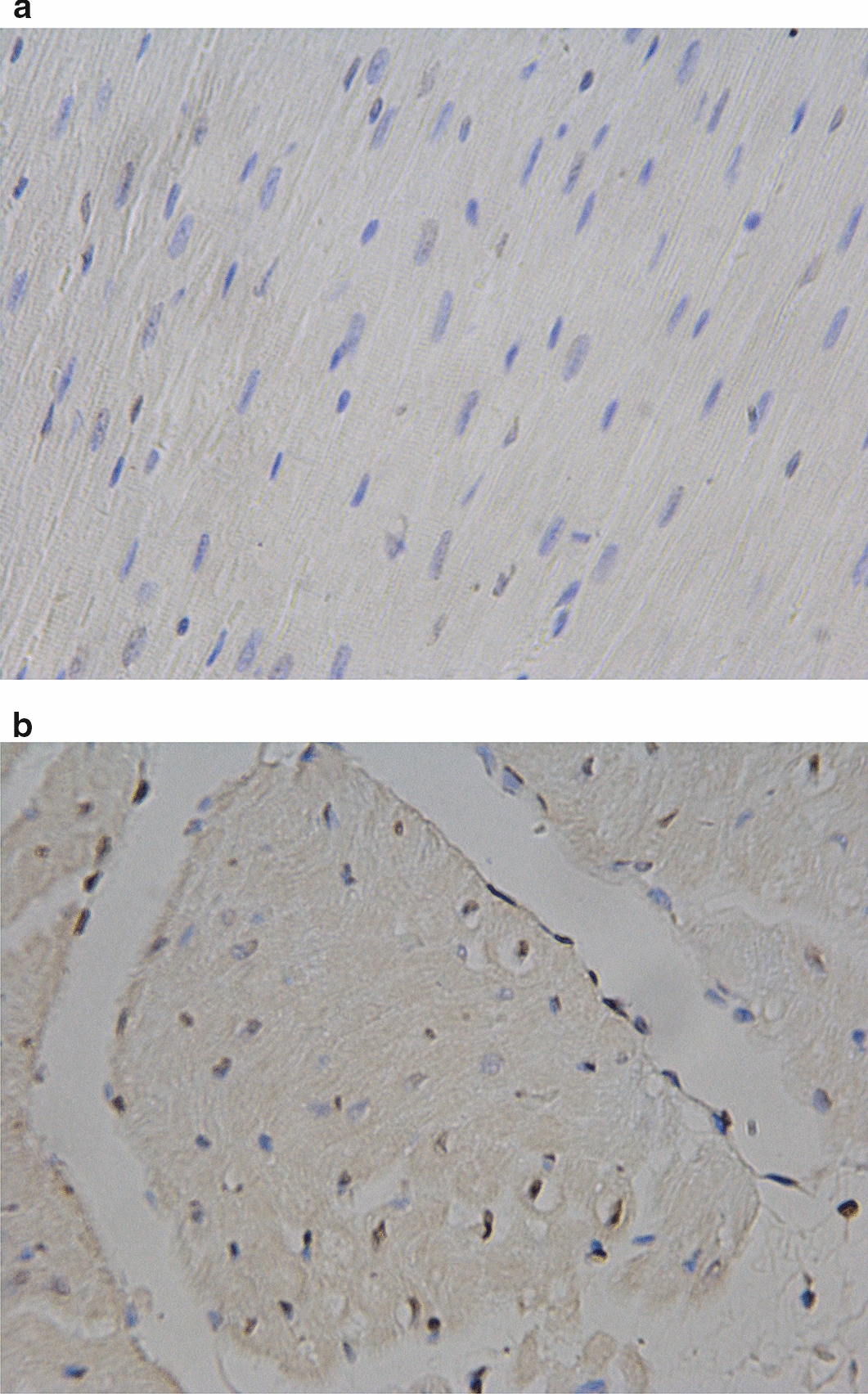
**a** TUNEL test showing manifestation of myocardial apoptotic cells (blue color) in control. **b** TUNEL test showing few manifestations of myocardial apoptotic cells (blue color) with vasodilation of intramyocardial vessels in tested dog (asphyxia model) after 2 h of device pulsations [23]

Two animals survived the CPR maneuver: 1 piglet, expired rapidly and excluded from the experiment, and 1 dog, kept alive for 6 h and served as a control. In the CFR group, there were spontaneous returns of heartbeats almost instantaneously as soon as the device began operating in two piglets whose hearts stopped for 8 min. In other animals with a longer cardiac arrest period (20–30 min), the device continued to operate for 2 h without return of heartbeats. There were significant improvements of hemodynamics data as depicted in Fig. 5a–d, the device induced a nearly physiological aortic pressure curve with a systolic pressure greater than 100 mmHg and carotid echo Doppler greater than 300 m/s. There was restoration of renal function with massive urine output in all animals within 15 min of device pulsations and improvement of peripheral cutaneous microcirculation. There was global vasodilation, compensated by IV fluids (1–2 L). The TUNEL test showed inferior apoptotic cells in the treated dog as well as an obvious dilation of the intracardiac coronary bed (Fig. 6a, b). The S100 protein serology test at T3 (Table 1) was lower in CFR treated dog compared to control: 0.39 ± 0.003 versus 0.48 ± 0.02 ng/mL, respectively.

**Table 1:** Results of S100 protein serology test (ng/mL)

Groups	T1	T2	T3
CFR	0.19 ± 0.01	0.25 ± 0.02	0.39 ± 0.003
CPR	0.22 ± 0.02	0.30 ± 0.01	0.48 ± 0.02

CFR treated dog after 30 min of cardiac arrest (asphyxia) + IV fluids for 2 h without return of heartbeat. CPR treated dog (chest compression + adrenaline + electric shocks) and maintained alive for 6 h. T1: baseline; T2; after arrest before treatment; T3: end of experiment (before sacrifice). S100 protein: a detector of brain damage

## Discussion

*Three* fundamental prerequisites suitable for ARDS management can be clearly deduced from the results of the present study, namely, the restoration and maintenance of ESS, along with the induction of adequate arterial pressure curve without vasopressors and tissue oxygenation without ventilators. For the first time in the literature, a low-pressure extracorporeal pulsatile device could induce a nearly physiological arterial pressure curve in cardiac arrest models (Fig. 5c, d), regardless of heartbeat. This means that the device’s vest, which is also served as a non-invasive mechanical ventilator, provided efficient cardiac compressions and recoil of chest wall and promoted ESS of the respiratory pump in two types of animal models with different cardiotorsal anatomies, compared to humans, e.g., more obtuse sternocostal angles and diaphragmatic attachments. Similarly, the alternating pulsations delivered by the infradiaphragmatic element at the stagnant hepatic-splanchnic venous capacitance increased RV preload, during the vest inspiratory phase, and decreased pulmonary afterload, manifested clinically by the improvements of hemodynamics, the cutaneous microcirculation, and urine output with significant vasodilation, which required compensation with IV fluids, despite the state of cardiac arrest. The improvement of microcirculation with ESS was demonstrated by lower myocardial apoptosis and dilated coronary bed, along with reduction S100 protein in the CFR treated dog (Fig. 5 and Table 1).

## ESS enhancement therapy in ARDS

### Pathophysiological evidence

The process of *ESS-microcirculation* interdependency constitutes the cornerstone of the proposed concept. While maintaining a full *respiratory pump* function, microcirculation behavior adapts to all circumstances of hematological disorders to ensure adequate tissue oxygenation by all means. For example, with a low or high hematocrit, the microcirculation exhibits a behavior that approximates that of Bernoulli’s law, as interpreted by the Fahraeus–Lindqvist effect [30], in which plasma stuck at the inner vascular boundary layers, while erythrocytes move faster at the center. This could explain the absence of cyanosis in anemic patients with low hematocrit, unlike those patients with high hematocrit, as erythrocytes aggregations at microcirculations induce cyanosis with clinical signs of clubbing fingers (drumsticks fingers). However, once the production of pulmonary endothelial mediators is compromised due to pathological conditions of contractile structures of the *respiratory pump,* e.g., ARDS, even with very mild hypoxia (SpO_2_ ≤ 94%), which is normally uncompromising for life, patients exhibit symptoms and signs,such as tachycardia, tachyarrhythmia, and orthopnea, which are pathophysiological accelerators of pulmonary ESS to improve microcirculation.

It is all about how to fully engage the respiratory pump and its influential forces, in particular, the gravitational effect to maintain the process of *ESS microcirculation* interdependency. For example, pulmonary ESS enhancement is the hallmark of physical exercise, exhibited without shortness of breath in marathon runners due to the “second wind” effect. In contrast, an overweight non-athlete runner exhibits shortness of breath and leans forward with hands on knees and not in a recumbent position. On the other hand, a congestive heart failure patient, in a recumbent position, exhibits a nocturnal orthopneic dyspnea to improve hemodynamics. Or patients in severe cyanosis (SpO_2_ ≤ 80%) exhibit squatting position without shortness of breath. This is exactly what lies behind the untold explanations of studies showing the advantages of non-invasive ventilation, low-dose neuromuscular blockade: maintaining chest wall recoil; prone position, obesities: in correlation with the diaphragmatic compression by the increased stagnant hepatosplanchnic venous capacity; tracheostomy: decreasing interalveolar pressure by reducing airflow energy losses and tracheal dead space.

### ESS enhancement vs. conventional therapies in ARDS

Three major clinical dilemmas must be resolved in critically ill ARDS patients, namely, PAH, increased stagnant venous capacity, and severe lack of tissue oxygenation. However, current PAH therapies whether with pharmacological and/or non-pharmacological supports, e.g., ECMO, ventilators, surgical procedures [31–35], remain insufficient with a dismal prognosis comparable with that of advanced cancer [36–38]. For example, inhalational nitric oxide (iNO) could increase endothelin-1 levels and decrease endogenous nitric oxide synthase (eNOS) activity [39]. Abrupt discontinuation of iNO can result in rebound PAH with further hemodynamic deterioration [40, 41]. Similarly, inhaled iloprost may cause acute bronchoconstriction [42–44], and the employment of CAD for PAH management is still linked with controversial results [45].

On the other hand, we have previously proved that PAH could be treated effectively with ESS induced by a new generation of pulsatile CAD to reduce PVR and improve hemodynamics in a nearly physiological manner and without pharmacological supports (Tables 2, 3) [46–48]. Also, in preclinical studies with a low-pressure pulsatile suit device, e.g., pulsatile trousers and mask prototypes, were tested on healthy volunteers, showed enhancement of the cutaneous microcirculation has also been observed, measured with a laser flowmeter (PeriFlux System 5000; Perimed) in an area remote from the pulsed zone (e.g., tip of the nose in mask trials, and fingertip with trousers) and increased cerebral blood flow (measured with carotid Doppler echo) after 20 min of un-synchronized pulsations (mask) [21, 49].

**Table 2:** Endothelial shear stress therapy versus conventional in cardiogenic shock models

Model	Surgical procedure	Pulsatile CAD	Control
Acute MI	Permanent LAD ligation	Intrapulmonary catheter	Nitrates
Acute PAH	Ao-pulm shunt	Intrapulmonary catheter	Tadalafil
Acute RVF	Pulm valve avulsion	Pulsatile trousers	Adrenaline, IV fluid, Tadalafil

*MI* myocardial ischemia, *PAH* pulmonary arterial hypertension, *RVF* right ventricular failure, *Ao-pulm* aortico-pulmonary artery shunt, *LAD* left anterior descending coronary artery, *Pulm* pulmonary (Refs. [46–48])

**Table 3:** Results of hemodynamic data of both groups: pulsatile and control (*n* = 36)

Pulsatile			Control	
Models	PVRI	CO	PVRI	CO
Acute MI	119 ± 13	0.92 ± 0.15	400 ± 42	0.52 ± 0.08
Acute PAH	85.8 ± 42.12	0.56 ± 0.26	478.6 ± 192.91	0.54 ± 0.11
Acute RVF	174 ± 60	1 ± 0.2	352 ± 118	0.7 ± 0.2

Groups: pulsatile (*n* = 18) and control (*n* = 18)

*MI* myocardial ischemia, *PAH* pulmonary arterial hypertension, *RVF* right ventricular failure, *PVRI* pulmonary vascular resistances index (dyne s/cm^−5^ kg^−1^), *CO* cardiac output (L/min)

*p* < 0.05 (2-way ANOVA)* (Refs. [46–48])

All these studies have demonstrated the undeniable crucial role of endogenous pulmonary endothelial mediators in controlling hemodynamics, microcirculation, and metabolism through the PVR, regardless of cardiac conditions, e.g., healthy, dysfunctional, or arrested.

The rapid reduction of PVR with hemodynamic improvements, proved the hypersensitivity of the unexploited right-heart side endothelium (e.g., pulmonary, venous, hepatic, etc.) to shear stress stimuli. For example, a few minutes of intrapulmonary catheter pulsations were more than enough to decrease a systolic pulmonary artery pressure (PAP) from ≥ 45 mmHg to approximately 9 mmHg within few minutes (≈ 10 min) and bit longer (≈ 20 min) with trousers pulsations. On the other hand, the most frequently stimulated left-heart side endothelium, e.g., intra-aortic balloon pump (IABP), shows a tolerance effect to ESS with further deterioration rather than restoration of endothelial function [50]. A nearly physiological effect that could be analogous to the natural phenomena observed with tetralogy of Fallot (TOF) when a patient with cyanotic spells assumes a squatting position to temporarily increase systemic vascular resistances (SVR) which increases intrapulmonary flow dynamics (ESS) through the overriding aorta to decrease PVR. Unlike vasopressors, which are largely used in acute PAH but with well-known complications, e.g., tachyarrhythmia, renal failure, etc. [51, 52].

We should remind that PAH is an endothelial dysfunction disease treated with pharmacological options which are functionally simulating what could be obtained naturally from the endothelium, but with side effects. These make restoration of endothelial function with ESS the optimal choice for PAH management.

**Fig. 7:**
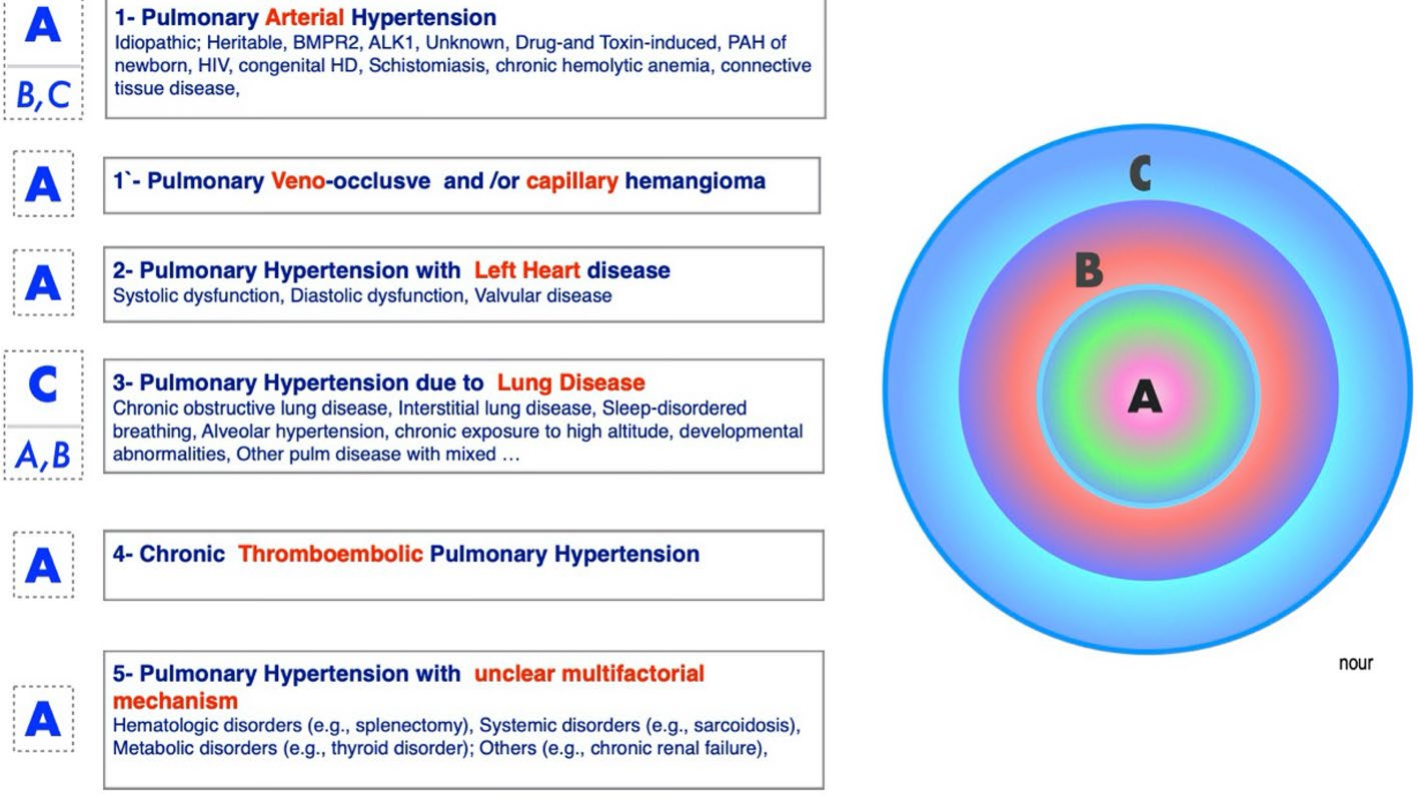
The ABC Target-Board Pulmonary Hypertension Management [22]. Right panel: schematic imaginary division of the human body into three rheological spheres (A, B, and C): A = amount of fluid; B = barriers of cells; C = covering tissues; “Somarheology theory” [23]. Middle panel: Dana Point PAH classification [53]. Left panel: mismatching the Dana Point groups of PAH & ABC spheres. Endothelial shear stress could be triggered at **B**, through **A** and/or **C**

Practically, a human body (Soma) can be divided into *three* imaginary hemorheological spheres [23]: **A**, **B**, and **C** (Somarheology theory), wherein A stands for the amount of fluids, that could be compressible Newtonian (e.g., air), or incompressible non-Newtonian (e.g., blood) fluids, surrounded by B, the barriers of cells (e.g., vascular endothelium, alveolar epithelium), overlapped by C, the covering tissues (e.g., vascular vessels, parenchyma, muscles, etc.). Therefore, reduction of PVR could be induced with a pulsatile device *internally* through sphere A and/or *externally*, e.g., through sphere C to create ESS at sphere B, and in correspondence to the Dana Point PAH classification [53], as depicted in Fig. 7 [22]. For example, in group C, e.g., Covid-19 patients with parenchymal congestion, delivery of ESS should be induced *externally* through sphere C with a pulsatile device adapted to patients’ pathophysiological requirements. As depicted in Fig. 3, the CFR device can induce ESS through several endothelial surrounding covering layers (C): parenchymal, mediastinal, thoracic cage muscles, and the diaphragm. As a result, the device stimulates the massive natural pulmonary and hepatic endothelial stocks, inducing plenty of mediators to restore hemodynamics and metabolic processes. Contrarily to NIV, which is only considered in the early stages of SARS, the device can be used effectively in severe ARDS [54]. Unlike the iron lung [55], it is a low-pressure pulsatile device (0.1–0.5 bar), that can mobilize both supra and infradiaphragmatic structures of the respiratory pump that makes it suitable for all ages and genders without side effects, e.g., rib fractures, mammary gland hematoma, etc. The device is an automated assembly that could be easily tilted at the request of clinicians, e.g., physiotherapists. Therefore, it could be an exclusive therapeutic tool for ARDS and achievement of current concept-based trials that showed some hope, but still remains without major impacts [56].

## Limitations

We have been confronted with some technical difficulties that include the use of two separate mal-synchronized pneumatic generators. A tissue prototype, which is less rigid at its outer part, makes the body compression less efficient, particularly, with the morphological difference between the dog and the pig that required specific prototypes for each model.

## Future prospects

We have planned to continue the development pathway of the CFR, as has been figured recently from the United States Food and Drug Administration [57], with preclinical studies for out of hospital cardiac arrest management. We have planned a PAH study in hypoxic piglets’ model. Both programs are in standby for logistic, unscientific reasons. Nevertheless, given the current pandemic with the shortage and/or controversial results of ventilators worldwide, in review with the FINER criteria for a good research question and the phases of evaluation of new therapies [58, 59], we consider a low-pressure non-invasive device is ease of manufacture and safe for use to promote endothelial shear stress to improve hemodynamics, tissues oxygenation, and metabolic processes, which significantly will improve the outcomes of critically ill Covid-19 patients.

## Conclusion

Compared with traditional therapies, ESS enhancement represents a more effective treatment to decrease PVR and improve hemodynamics in ARDS. This method could be induced properly with pulsatile CAD adaptable for pathophysiology and biophysics of *three* hemorheological spheres (ABC) that may assemble the several forms of the disease. This represents a cost-effective method and safer procedure compared to current therapies.

## Data Availability

Data are available from the corresponding author upon request.

## Abbreviations

ESS: Endothelial shear stress
PVR: Pulmonary vascular resistances
ARDS: Acute respiratory distress syndrome
SARS: Severe acute respiratory syndrome
ECMO: Extracorporeal membrane oxygenation
LV: Left ventricle
CAD: Circulatory assist device
BV: Blood volume
SpO_2_: Peripheral capillary oxygen saturation
CO: Cardiac output
NIV: Non-invasive ventilation
CFR: Circulatory flow restoration
CPR: Cardiopulmonary resuscitation
MPa: Megapascal
SCA: Sudden cardiac arrest
CVP: Central venous pressure
PAH: Pulmonary arterial hypertension
MI: Myocardial ischemia
RVF: Right ventricular failure
LAD: Left anterior descending coronary artery
IABP: Intra-aortic balloon pump
PAP: Pulmonary arterial pressure
SVR: Systemic vascular resistances
TOF: Tetralogy of Fallot
NO: Nitric oxide
iNO: Inhalational nitric oxide
FINER: Feasible, interesting, novel, ethical, and relevant

## Footnotes

1 For further details please refer to the patented descriptions: US20140148739.

## References

[1] Huxley TH (1870). Biogenesis and abiogenesis. Nature.

[2] LeDuc JW, Barry MA (2004). SARS, the first pandemic of the 21st Century. Emerg Infect Dis.

[3] Combes A, Hajage D, Capellier G (2018). Extracorporeal membrane oxygenation for severe acute respiratory distress syndrome. N Engl J Med.

[4] Ni W, Yang X, Yang D (2020). Role of angiotensin-converting enzyme 2 (ACE2) in COVID-19. Crit Care.

[5] Bikdeli B, Madhavan MV, Jimenez D (2020). COVID-19 and thrombotic or thromboembolic disease: implications for prevention, antithrombotic therapy, and follow-up. J Am Coll Cardiol.

[6] Li Y, Zheng J, Bird IM, Magness RR (2005). Effects of pulsatile shear stress on signaling mechanisms controlling nitric oxide production, endothelial nitric oxide synthase phosphorylation, and expression in ovine fetoplacental artery endothelial cells. Endothelium.

[7] Petrovic D, Zorc-Pleskovic R, Zorc M (2000). Apoptosis and proliferation of cardiomyocytes in heart failure of different etiologies. Cardiovasc Pathol.

[8] Nour S (2020). Deceptive slanders of cardiovascular pathology in Covid-19 ethnic minorities. Int J Integr Cardiol.

[9] Nour S, Liu J, Dai G (2014). Shear stress, energy losses and costs: a resolved dilemma of pulsatile cardiac assist devices. Biomed Res Int.

[10] Nour S, Wu G, Zhensheng Zh (2009). The forgotten driving forces in right heart failure: concept and device. Asian Cardiovasc Thorac Ann.

[11] Slaughter MS (2010). Long-term continuous flow left ventricular assist device support and end-organ function: prospects for destination therapy. J Card Surg.

[12] Giglia TM, Humpl T (2010). Preoperative pulmonary hemodynamics and assessment of operability: is there a pulmonary vascular resistance that precludes cardiac operation?. Pediatr Crit Care Med.

[13] Koh TJ, DiPietro LA (2011). Inflammation and wound healing: the role of the macrophage. Expert Rev Mol Med.

[14] Samet I, Lelkes PI (1999). Mechanical forces and endothelium.

[15] Neri Serneri GG (1981). Pathophysiological aspects of platelet aggregation in relation to blood flow rheology in microcirculation. Ric Clin Lab.

[16] Koller A, Kaley G (1990). Endothelium regulates skeletal muscle microcirculation by a blood flow velocity-sensing mechanism. Am J Physiol.

[17] SOS-KANTO study group (2009). Comparison of arterial blood gases of laryngeal mask airway and bag-valve-mask ventilation in out-of-hospital cardiac arrests. Circ J.

[18] Cooper JA, Cooper JD, Cooper JM (2006). Cardiopulmonary resuscitation: history, current practice, and future direction. Circulation.

[19] Ioannidis G, Lazaridis G, Baka S (2015). Barotrauma and pneumothorax. J Thorac Dis.

[20] Grillo F, Barisione E, Ball L (2020). Lung fibrosis: an undervalued finding in COVID-19 pathological series. Lancet Infect Dis.

[21] Nour S. New hemodynamic theory “Flow and Rate”: concept and clinical applications using new pulsatile circulatory assist devices. Ph.D. Thesis, Therapeutic Innovations, University Paris Sud, Paris XI; Français. 2012. NNT: 2012PA114862.

[22] Nour S (2014). Fiction mirrors truth! The ABC target-board for pulmonary arterial hypertension. C53. Clinical evaluation and biomarkers of pulmonary hypertension II.

[23] Nour S, Carbognani D, Chachques JC (2017). Circulatory flow restoration versus cardiopulmonary resuscitation: new therapeutic approach in sudden cardiac arrest. Artif Organs.

[24] Olufsen MS, Nadim A (2004). On deriving lumped models for blood flow and pressure in the systemic arteries. Math Biosci Eng.

[25] Roselli RJ, Brophy SP (2003). Redesigning a biomechanics course using challenge-based instruction. IEEE Eng Med Biol Mag.

[26] Brettner F, Chappell D, Schwartz L (2017). Vascular endothelial dysfunction during cardiac surgery: on-pump versus off-pump coronary surgery. Eur Surg Res.

[27] Davison SN (2003). Pain in hemodialysis patients: prevalence, cause, severity, and management. Am J Kidney Dis.

[28] Botran M, Urbano J, Solana M (2010). 56 cardiopulmonary resuscitation by chest compressions versus ventilation plus chest compressions in a pediatric asphyxial cardiac arrest animal model. Pediatr Res.

[29] Sun B-d, Liu H-M, Nie S-N (2013). S100B protein in serum is elevated after global cerebral ischemic injury. World J Emerg Med.

[30] Fahraeus R, Lindquist T (1931). The viscosity of the blood in narrow capillary tubes. Am J Physiol.

[31] Boutet K, Montani D, Jaïs X (2008). Review: therapeutic advances in pulmonary arterial hypertension. Ther Adv Respir Dis.

[32] Checchia PA, Bronicki RA, Goldstein B (2012). Review of inhaled nitric oxide in the pediatric cardiac surgery setting. Pediatr Card.

[33] Tessler RB, Zadinello M, Fiori H (2008). Tadalafil improves oxygenation in a model of newborn pulmonary hypertension. Pediatr Crit Care Med.

[34] Nemoto S, Sasaki T, Ozawa H (2010). Oral sildenafil for persistent pulmonary hypertension early after congenital cardiac surgery in children. Eur J Cardiothorac Surg.

[35] McLaughlin VV, Benza RL, Rubin LJ (2010). Addition of inhaled treprostinil to oral therapy for pulmonary arterial hypertension: a randomized controlled clinical trial. J Am Coll Cardiol.

[36] Humbert M, Morrell NW, Archer SL (2004). Cellular and molecular pathobiology of pulmonary arterial hypertension. J Am Coll Cardiol.

[37] Felker GM, Thompson RE, Hare JM (2000). Underlying causes and long-term survival in patients with initially unexplained cardiomyopathy. N Engl J Med.

[38] Nef HM, Möllmann H, Hamm C (2010). Pulmonary hypertension: updated classification and management of pulmonary hypertension. Heart.

[39] Macchia A, Marchioli R, Marfisi R (2007). A meta-analysis of trials of pulmonary hypertension: a clinical condition looking for drugs and research methodology. Am Heart J.

[40] Bush A (2006). Pulmonary and critical care updates (update in pediatrics 2005). Am J Respir Crit Care Med.

[41] Oishi P, Grobe A, Benavidez E (2006). Inhaled nitric oxide induced NOS inhibition and rebound pulmonary hypertension: a role for superoxide and peroxynitrite in the intact lamb. Am J Physiol Lung Cell Mol Physiol.

[42] Miller OI, Tang SF, Keech A (1995). Rebound pulmonary hypertension on withdrawal from inhaled nitric oxide [letter]. Lancet.

[43] Segal ES, Valette C, Oster L (2005). Risk management strategies in the postmarketing period: safety experience with the US and European Bosentan Surveillance Programmes. Drug Saf.

[44] Stevens T (2008). Lung vascular cell heterogeneity: endothelium, smooth muscle, and fibroblasts. Proc Am Thorac Soc.

[45] Martin J, Siegenthaler MP, Friesewinkel O (2004). Implantable left ventricular assist device for treatment of pulmonary hypertension in candidates for orthotopic heart transplantation—a preliminary study. Eur J Cardiothorac Surg.

[46] Nour S, Dai G, Carbognani D (2012). Intrapulmonary shear stress enhancement: a new therapeutic approach in pulmonary arterial hypertension. Pediatr Cardiol.

[47] Nour S, Yang D, Dai G (2013). Intrapulmonary shear stress enhancement: a new therapeutic approach in acute myocardial ischemia. Int J Cardiol.

[48] Nour S, Dai G, Wang Q (2012). Forgotten driving forces in right heart failure (Part II): experimental study. Asian Cardiovasc Thorac Ann.

[49] Nour S, Misra AN (2012). Flow and rate: concept and clinical applications of a new hemodynamic theory, Ch. 17–76. Biophysics.

[50] Scholz KH (1999). Reperfusion therapy and mechanical circulatory support in patients in cardiogenic shock. Herz.

[51] Thomas M (2008). Management of pulmonary hypertension in the intensive care unit. Crit Care Med.

[52] Zamanian RT, Haddad F, Doyle RL, Weinacker AB (2007). Management strategies for patients with pulmonary hypertension in the intensive care unit. Crit Care Med.

[53] Simonneau G, Robbins IM, Beghetti M (2009). Updated clinical classification of pulmonary hypertension. JACC.

[54] Lau AC, Yam LY, So LK (2004). Management of critically ill patients with severe acute respiratory syndrome (SARS). Int J Med Sci.

[55] Goti P, Duranti R, Spinelli A (1995). Effects of the iron lung on respiratory function in chronic hypercapnic COPD patients. Arch Chest Dis.

[56] Scholten EL, Beitler JR, Prisk GK (2017). Treatment of ARDS with prone positioning. Chest.

[57] Guan A, Hamilton P, Wang Y (2017). Medical devices on chips. Nat Biomed Eng.

[58] Hulley SB, Cummings SR, Browner WS (2013). Designing clinical research.

[59] Pocock SJ, Clayton TC, Stone DW (2015). Challenging issues in clinical trial design Part 4 of a 4-part series on statistics for clinical trials. J Am Coll Cardiol.

